# An Automated Comparative Observation System for Sun-Induced Chlorophyll Fluorescence of Vegetation Canopies

**DOI:** 10.3390/s16060775

**Published:** 2016-05-27

**Authors:** Xijia Zhou, Zhigang Liu, Shan Xu, Weiwei Zhang, Jun Wu

**Affiliations:** 1 State Key Laboratory of Remote Sensing Science, School of Geography, Beijing Key Laboratory of Environmental Remote Sensing and Digital City, Beijing Normal University, Beijing 100875, China; zxj1992@139.com (X.Z.); hzauxushan@126.com (S.X.); zhangww0510@163.com (W.Z.); 2 Guangxi Colleges and Universities Key Laboratory of Optoelectronic Information Processing, Guilin University of Electronic Technology, Guilin 541004, Guangxi, China; wujun93161@hotmail.com

**Keywords:** remote sensing of vegetation, sun-induced chlorophyll fluorescence, vegetation canopy, comparative observation, automatic observation, FluorMOD model

## Abstract

Detecting sun-induced chlorophyll fluorescence (SIF) offers a new approach for remote sensing photosynthesis. However, to analyse the response characteristics of SIF under different stress states, a long-term time-series comparative observation of vegetation under different stress states must be carried out at the canopy scale, such that the similarities and differences in SIF change law can be summarized under different time scales. A continuous comparative observation system for vegetation canopy SIF is designed in this study. The system, which is based on a high-resolution spectrometer and an optical multiplexer, can achieve comparative observation of multiple targets. To simultaneously measure the commonly used vegetation index and SIF in the O_2_-A and O_2_-B atmospheric absorption bands, the following parameters are used: a spectral range of 475.9 to 862.2 nm, a spectral resolution of approximately 0.9 nm, a spectral sampling interval of approximately 0.4 nm, and the signal-to-noise ratio (SNR) can be as high as 1000:1. To obtain data for both the upward radiance of the vegetation canopy and downward irradiance data with a high SNR in relatively short time intervals, the single-step integration time optimization algorithm is proposed. To optimize the extraction accuracy of SIF, the FluorMOD model is used to simulate sets of data according to the spectral resolution, spectral sampling interval and SNR of the spectrometer in this continuous observation system. These data sets are used to determine the best parameters of Fraunhofer Line Depth (FLD), Three FLD (3FLD) and the spectral fitting method (SFM), and 3FLD and SFM are confirmed to be suitable for extracting SIF from the spectral measurements. This system has been used to observe the SIF values in O_2_-A and O_2_-B absorption bands and some commonly used vegetation index from sweet potato and bare land, the result of which shows: (1) the daily variation trend of SIF value of sweet potato leaves is basically same as that of photosynthetically active radiation (PAR); and (2) the bare land is a non-fluorescent emitter, the SIF of which is significantly smaller than that of sweet potato; and (3) analysis result based on the measured data is basically same as that based on simulated data. The above results verified the reliability of the SIF extracted from the measured data and the feasibility of comparatively observing the SIF value and the commonly used vegetation index of multiple vegetation canopy with this continuous observation system. This approach is beneficial for comprehensively analysing the stress response characteristics of vegetation canopies.

## 1. Introduction

As the fluorescence emission of chlorophyll in vegetation is closely related to the photosynthesis occurring in the leaves [1], information about photosynthesis can be obtained by detecting chlorophyll fluorescence. Accordingly, sun-induced chlorophyll fluorescence (SIF) may provide an effective means for detecting stress in vegetation. Indeed, some progress has been made with regard to water stress. For example, a water-stress experiment by Zarco-Tejada *et al.* showed that the chlorophyll fluorescence computed in a hyperspectral image is correlated with the stomatal conductance and water potential of orange leaves [2]. Lee *et al.* extracted SIF data of the Amazon Basin from GOSAT satellite data and showed that SIF and ground water are both reduced at noon during the dry season [3]. In addition, the canopy water content is highly correlated with the canopy SIF at the time scale of a month. Regardless of the potential revealed by the preliminary research, the response regularity of SIF in the red and near-infrared bands for stress conditions of different vegetation at different time scales remains unclear. Thus, further canopy observation experiments are required [4]. Because SIF is largely affected by external environmental factors, it offers an effective experimental approach to synchronizing observations of vegetation with different degrees of stress.

Multiple sets of continuous spectral observation systems for vegetation have been designed worldwide, largely by the EUROSPEC Cost Action ES0930 project [5]. These observation systems can be divided into the following two types according to the number of spectrometers. The first type is composed of single-spectrometer continuous observation systems. Typical systems of this type include the Multiplexer Radiometer Irradiometer (MRI) designed by Cogliati *et al.* [6] and the HyperSpectral Irradiometer designed by Meroni *et al.* and Rossini *et al.* [7,8,9]. This type of system requires a fibre-optic splitter or mechanical rotating device to obtain successive measurements of the incident downward radiance and upward radiance. Because there is a specific time interval between these two measurements, the change in irradiance during this period will influence the SIF extraction accuracy. The second type is composed of double-spectrometer continuous-observation systems. A typical system of this type is the continuous observation system designed by Drolet *et al.* [10]. The main restriction of this type of system is that the spectrometers need to conduct a frequent inter-calibration because spectrometer performance changes with time, temperature and other factors. The above systems have two general limitations. First, these systems mainly consider how to perform an automatic continuous observation of SIF for a certain vegetation canopy and cannot achieve comparative observations for different vegetation canopies. Therefore, they are not convenient for observing the response characteristics of SIF under different stress states. Second, these systems mainly consider SIF extraction in the O_2_-A absorption band. However, because the spectral resolution and signal-to‑noise ratio (SNR) are not adequate, the above systems cannot accurately extract SIF in the O_2_-B absorption band. Related studies have shown that both red and far-red SIF would be beneficial for detecting plant stress events and changes in both the chlorophyll concentration and plant canopy structure [4,11].

To further improve the above deficiency and lower the cost as much as possible, existing continuous observation systems are improved in this research. The system is based on a single QE65pro spectrometer and an MPM-2000 optical multiplexer (Ocean Optics Inc., Dunedin, FL, USA), which can be expanded to observe multiple targets, for example, observation of multiple vegetation types under different stresses. To synchronously measure the commonly used vegetation index and SIF in both the O_2_-A and O_2_-B absorption bands, the spectral response range of this system is 475.9 to 862.2 nm. To ensure the best possible SNR and to shorten the time interval between the measurement of downward irradiance and upward radiance, the study improves upon the strategy of optimizing the integration time presented by Cogliati *et al.* [6]. In this research, a single-step integration time optimization algorithm is used, which can obtain the high SNR data of the canopy upward radiance and downward irradiance in succession in a short time interval, thus guaranteeing the reliability of the SIF extraction. The specific configuration of this system, the optimization method of the measured spectral data, the optimization method of the accuracy of each fluorescence extraction and the results of field observations are introduced and analysed in this paper.

## 2. Materials and Methods

### 2.1. Automatic Observation System of the Vegetation Canopy

**Figure 1:**
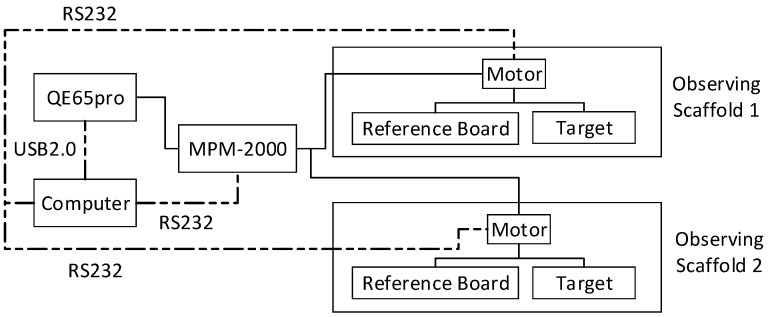
The structure of the automatic observation system.

The structure of the automatic observation system is shown in Figure 1 and includes a spectrometer, an MPM-2000 optical multiplexer, two scaffolds for observation of the vegetation canopy and a control computer. The spectrometer in this system is the QE65pro spectrometer of Ocean Optics, which has a spectral range of 475.921 to 862.227 nm, a spectral resolution of approximately 0.9 nm, and a spectral sampling interval of approximately 0.4 nm. The sensitivity of the QE65pro spectrometer is very high. Moreover, this spectrometer includes a refrigeration system, which can decrease the internal temperature to approximately 40 °C below ambient, thereby sharply lowering the dark current and dark noise. The maximum SNR of this spectrometer can reach 1000:1. The MPM-2000 optical multiplexer can link the spectrometer up to eight channels and can switch between different channels in less than 150 milliseconds. Therefore, the spectrometer can simultaneously observe up to 8 targets. In this present observation system, the spectrometer is connected with two channels, and the optical fibre of each channel is fixed on a separate observation scaffold. An electrical device is used to rotate the optical fibre probe to switch between the measuring target and reference board. The control program of this system operates the spectrometer, the MPM-2000 and the electrical rotation device.

**Figure 2:**
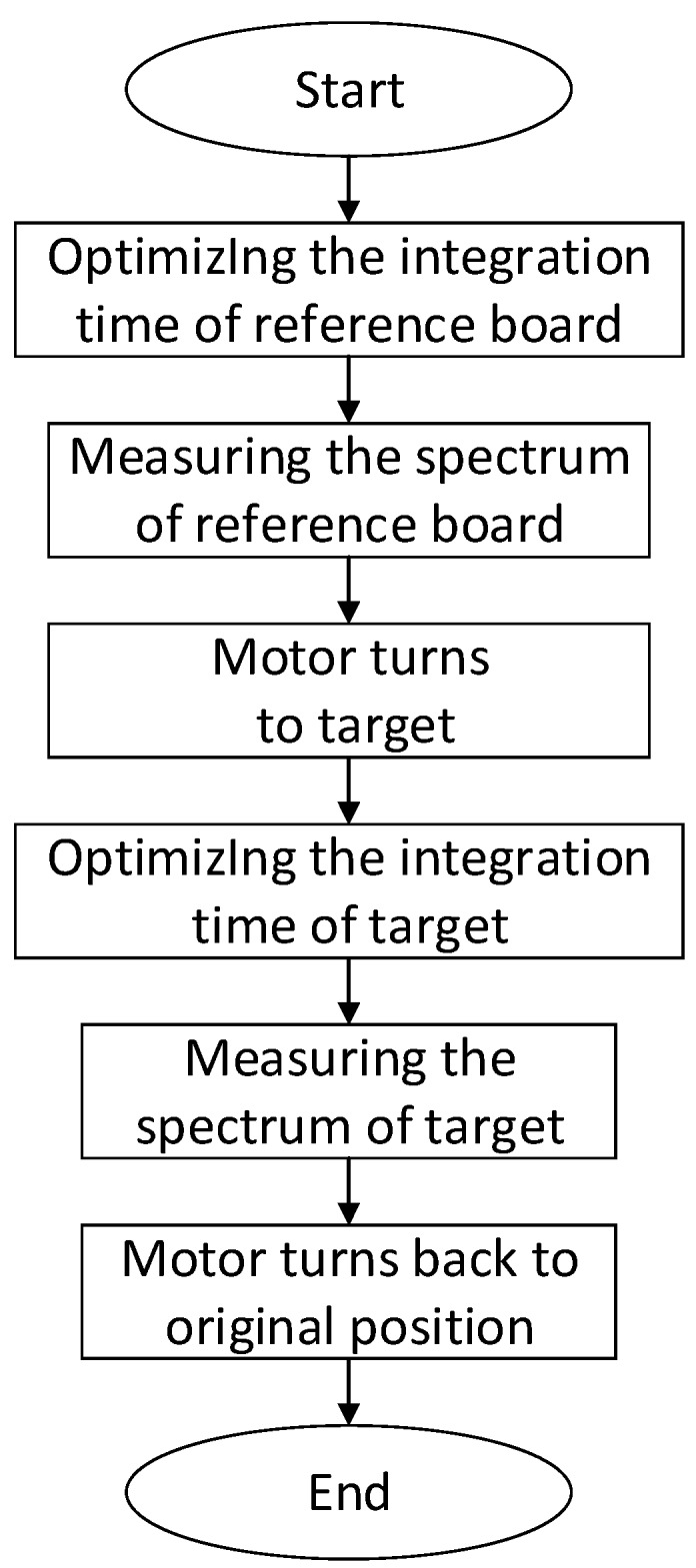
Flowchart of a single measurement process for the vegetation canopy continuous observation system.

Obtaining a group of SIF measurements includes the follow procedures: (1) integration time optimization of the reference board; (2) measurement of the reference board’s spectrum; (3) integration time optimization of the measuring target; and (4) measurement of the measuring target’s spectrum (Figure 2). To improve the SNR as much as possible, the measurement integration time must be optimized. Moreover, the luminance difference of the reference board and measuring target is very large; therefore, the integration time of the reference board and the measuring target needs to be optimized. To shorten the time needed for optimizing the integration time as much as possible, a single‑step integration time optimization algorithm is used in this research, which is different from that of Cogliati *et al.* [6]. Assuming that the change in the spectrometer’s response within certain ranges of light intensity is linear, the optimized integration time can be computed by the follow formula:

(1) $$IT_{Opt}=IT_{init}\times \frac{Counts_{Opt}}{Counts_{init}}$$

where $Counts_{Opt}$ is the count value of the spectrometer corresponding to the optimal integration time, which is generally set to 80%~85% of the saturated count values (approx. 65,000). At this value, the degree of linearity of the signal response of the spectrometer is relatively high. $IT_{init}$ is the initial integration time. $Counts_{init}$ is the maximum count value of the spectral curve at the initial integration time, from which the dark current has been subtracted. Because the saturated count value of the QE65pro spectrometer is much larger than the dark current (no more than 3000 in this research), the optimized spectral value cannot exceed the saturation value, even though the dark current is not subtracted from the saturated count values. Therefore, a satisfactory result can be achieved when using Equation (1) to optimize the integration time for less than one second at local solar noon. $IT_{Opt}$ is the optimized integration time. In this research, the maximum count value corresponding to the optimized integration time essentially remains at approximately 50,000, which is approximately 80% of the saturated count value. After optimizing the integration time, the measurement of the spectrum of the reference plate begins. The optical fibre probe is then adjusted either toward the vegetation canopy or toward bare land by turning the motor. Next, the measuring target integration time is optimized by applying the above method, and the target spectrum is measured. Finally, the motor is returned to its original position and waits for the next round of measurements.

### 2.2. Simulated Data

To optimize the extraction precision of SIF with a certain spectral resolution, sampling interval and SNR of the QE65pro spectrometer in this research, the atmospheric radiation transfer model MODTRAN4 and the vegetation canopy fluorescence model FluorMOD [12,13] are used to simulate a set of data that include different vegetation parameters. These simulated data are used to analyse the optimal parameters and the precision of SIF of each algorithm in the O_2_-A and O_2_-B absorption bands.

#### 2.2.1. Simulation of Canopy Spectral Data

##### Simulation of Spectral Data under Different Parameters of the Canopy and Leaves

**Figure 3:**
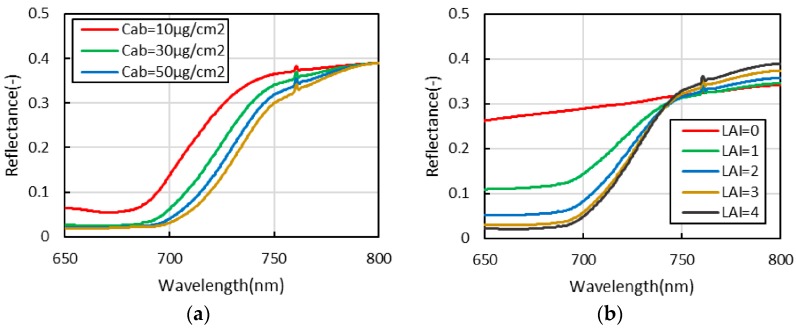
The vegetation reflectance spectrum with different chlorophyll contents and leaf area indices. (**a**) The vegetation reflectance spectrum with different chlorophyll contents; (**b**) the vegetation reflectance spectrum with different leaf area indices.

The chlorophyll content and leaf area index are the major factors that determine the shape of the vegetation canopy reflectance in the oxygen absorption bands, thus affecting the precision of fluorescence extraction. Therefore, to analyse the fluorescence extraction accuracy of each algorithm for data with a different chlorophyll content and leaf area index, the chlorophyll content is set to 10, 30, 50 and 70 μg/cm^2^; the leaf area index is set to 0, 1, 2, 3 and 4; and the sun zenith angle is set to 30°. The chlorophyll content is the mass of chlorophyll per square meter, the leaf area index is the total blade area per square meter of land, and the sun zenith angle is the angle of incoming light and local zenith direction. The other parameters are kept at the default settings. The specific parameter settings are shown in Table 1 and Table 2. Figure 3 illustrates the vegetation reflectance spectrum for different chlorophyll contents and leaf area indices. In the O_2_-A absorption band, the chlorophyll content positively correlates with the slope of the trend line of the reflectance curve. The influence of the change in the leaf area index to the slope of the reflectance spectrum is smaller than that of the chlorophyll content. In the O_2_-B absorption band, the chlorophyll content is negatively correlated with the curvature of the reflectance spectrum and the slope of the trend line. Additionally, the leaf area index is positively correlated with the curvature of the reflectance curve.

##### Simulation of Spectral Data under Different Solar Zenith Angles

To analyse the accuracy of each fluorescence extraction algorithm under different solar zenith angles and to examine the accuracy of the fluorescence extraction from measured data, the spectral data of vegetation and bare land under different solar zenith angles are simulated in this research. The chlorophyll concentration of the vegetation is 40 μg/cm^2^, the leaf area index is 4, the sun zenith angle is between 10° and 70°, and the interval is 5°. The spectra of the simulated vegetation show slight changes under different solar zenith angles due to the sunlit area of the vegetation canopy, shaded area, and the soil background signal within the scope of observation. However, the spectra of bare land do not change under different solar zenith angles.

#### 2.2.2. Simulation of the Spectral Resolution and Spectral Sampling Interval

The FluorMOD model can simulate vegetation canopy reflectance and fluorescence within a range of 400 to 1000 nm with a spectral resolution of 1 nm. Because the QE65pro spectrometer in this research has a higher spectral resolution and spectral sampling interval, the MODTRAN4 model is first used to simulate the solar downward radiance, of which the spectral sampling interval is 1 cm^−1^ (the wavelength interval near 700 nm is approximately 0.05 nm), to simulate data with higher spectral resolutions and spectral sampling intervals. Next, those data are multiplied by the real reflectivity of the vegetation after resampling and added to the chlorophyll fluorescence data. The apparent upward radiance data of the vegetation is thus obtained, of which the wavelength interval is approximately 0.05 nm.

To simulate the spectral resolution and spectral sampling interval of the QE65pro spectrometer in this research, the simulated data are processed using Gaussian smoothing and resampling according to the spectral resolution and the spectral sampling interval of the spectrometer. Referencing Damm *et al.* [14], the simulated data are first subjected to Gaussian smoothing according to the spectral resolution of the QE65pro spectrometer. Next, resampling of the above result is performed according to the wavelength position and the spectral sampling interval of the QE65pro spectrometer. The filter kernel of Gaussian smoothing can be calculated by the following formula:

(2) $$K\left(\lambda \right)=\frac{2\sqrt{2ln2}}{\sqrt{2\pi \cdot }\sqrt{FWHM_{d}^{2}-SSI_{o}^{2}}}\cdot \exp\left(-\frac{4(ln2)\lambda^{2}}{FWHM_{d}^{2}-SSI_{o}^{2}}\right)$$

where λ is the wavelength of the filter kernel, FWHM is Full Width Half Maximum (*i.e.*, spectral resolution), SSI is the spectral sampling interval, SSI_o_ is the spectral sampling interval before smoothing, and FWHM_d_ is the spectral resolution after smoothing. To cause the spectral sampling interval to remain the same at all wavelengths, first the spectral sampling interval of the original data is resampled at a resolution of 0.01 nm. Next, the wavelength interval of the filter kernel is set to 0.01 nm, and the wavelength variation interval is set to the FWHM after filtering (*i.e.*, 0.9 nm). Therefore, the wavelength range is [−0.45, 0.45], and the size of the filter kernel is 91.

#### 2.2.3. Simulation of Noise

To simulate the influence of the random noise of the QE65pro spectrometer on the fluorescence extraction accuracy, noise is further added to each wavelength of the simulated spectral curve; thus, 1000 solar downward radiance curves and 1000 target upward radiance curves including noise are generated. The noise added is calculated according to the Gaussian distribution; the mean value is 0, and the standard deviation is the ratio of the radiance to the SNR. Because the magnitude of the radiance differs at different wavelengths, the actual SNR at different wavelengths is different. Equation (3) [15] is used to calculate the actual SNR at each wavelength. According to the description file, the SNR corresponding to the saturation value of the spectrometer is assumed to be 1000:1, but the ratio of the SNR to the square root of the count value of the spectrometer remains unchanged within the scope of the entire band:

(3) $$SNR\left(\lambda \right)=SNR_{ref}\cdot \sqrt{\frac{counts\left(\lambda \right)}{counts_{ref}}}$$

where $counts_{ref}$ is the referenced count value, $SNR_{ref}$ is the SNR corresponding to the referenced count value, SNR(λ) is the SNR at a certain wavelength, and counts(λ) is the count value at a certain wavelength. To compute the count value of the simulated data, the radiance of the simulated data is first divided by the calibration coefficient of the QE65pro spectrometer. The maximum of the above result is then stretched to 50,000, which is approximately 80% of the spectrometer’s saturation value. If the dark noise (only approx. 4 counts) of the spectrometer is ignored, then the formula for adding noise can be shown as:

(4) $$L_{noise}(\lambda )=L(\lambda )+\left(noise\times \frac{L(\lambda )}{SNR(\lambda )}\right)$$

where $L_{noise}(\lambda )$ is the simulated data after adding noise, L(λ) is the data without noise, noise is the ratio of actual noise to the greatest noise, which obey the standard Gaussian distribution, and SNR(λ) is the SNR at a certain wavelength [14].

### 2.3. Experimental Scheme

The Fraunhofer Line Depth (FLD), Three FLD (3FLD) and spectral fitting method (SFM) [16,17] are used to extract the fluorescence in the O_2_-A and O_2_-B absorption bands. To optimize the fluorescence extraction accuracy of the ground experimental data, the simulated data are first used to analyse the best parameters and accuracy of each algorithm. These algorithms and their best parameters are then used to extract the fluorescence from the ground experimental data. To test the optimized effect of each fluorescence extraction algorithm, the accuracy of each algorithm after parameter optimization is compared by extracting the fluorescence using other parameters. The parameters set of each fluorescence extraction algorithm are shown in Table 3 and Table 4. Each algorithm has 3 types of parameters set. Because 3FLD assumes that the fluorescence at the nearby atmospheric absorption band remains unchanged, the distance of the wavelengths on both sides of the absorption band to the bottom of the absorption band is equal among the several groups of 3FLD parameters selected in this research. For SFM, 3 types of bands with different wavelength scopes are used to fit reflectance and fluorescence.

**Figure 4:**
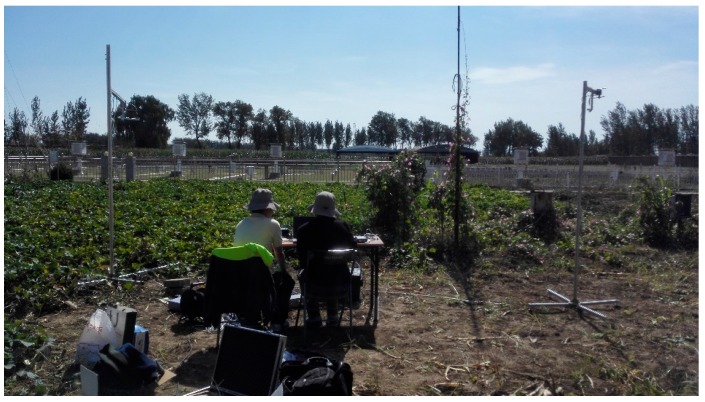
The comparative observation of sweet potato and bare land.

This system was used to observe the diurnal variation of spectra for sweet potato and bare land on 12 October 2015 (Figure 4). The research area is in Baoding, Hebei Province, China (39°8’40.38”N; 115°44’16.25”E). The measuring time is 10:30 to 16:30 Beijing time, and the cloud cover is insignificant during the entire observation. This system obtains a group of data for sweet potato once per minute. Because bare land can be viewed as a not emitting fluorescence, the purpose of observing bare land is to test the extraction accuracy of SIF; thus, the counts of bare land observation are relatively low. To further reduce the noise of the data and improve the accuracy of the fluorescence extraction algorithm, each wavelength is averaged with three adjacent wavelengths. Moreover, the measured reflectance spectra of the vegetation can be used to compute vegetation indices such as the Red-Edge Chlorophyll Index [18] and the Photochemical Reflectance Index (PRI) [19]. At the same time as the spectral measurement, the LI-1500 Light Sensor Logger is used to obtain the photosynthetically active radiation (PAR) once per minute.

## 3. Results and Analysis

### 3.1. Analysis of Simulation Data

#### 3.1.1. Selection of Algorithm Parameters

Through repeated trials of different parameters of each algorithm and referencing the method of Meroni *et al.* [16], the settings for each parameter in the O_2_-A and O_2_-B absorption bands are summarized in Table 5 and Table 6. For data containing noise, the wavelengths at either side of the absorption band need to be set to a position relatively far from the bottom of the absorption band, and to reduce the influence of noise on the fluorescence extraction results, the fitting function of SFM must be a polynomial function with a relatively low degree.

**Figure 5:**
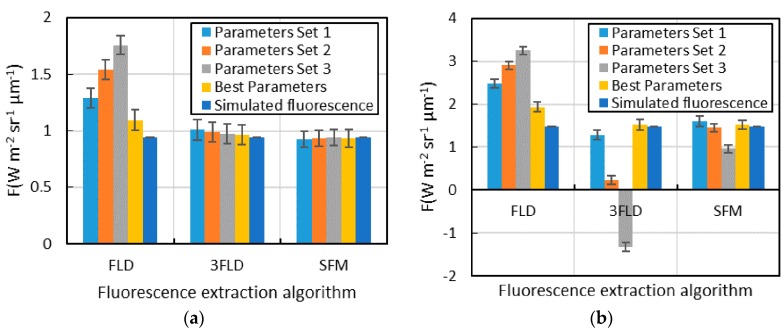
The fluorescence extraction results for each algorithm under different parameters set and the simulated value of fluorescence (the rectangle in this figure is the mean fluorescence, and the error lines represent the standard deviation); (**a**) O_2_-A absorption band; (**b**) O_2_-B absorption band.

Taking the simulation with a chlorophyll content of 40 and with a Leaf area index of 4 as an example, the parameter optimization effect of each fluorescence extraction algorithm is tested (Figure 5). The different parameters set for the test of the optimization effect have been shown in Section 2.3. In the O_2_-A absorption band, the fluorescence extraction accuracy of FLD clearly changes under the different parameters, whereas the accuracy of 3FLD and SFM do not show obvious changes under the parameters in this study. In the O_2_-B absorption band, the accuracy of every algorithm changes to a different degree under different parameters, with the accuracies of FLD and 3FLD showing large variations and that of SFM showing a relatively small variation. The variation in the accuracy of each algorithm in the O_2_-B absorption band is larger than that in the O_2_-A absorption band. This may be because the shape of the reflectance curve cannot be expressed using a straight line and the reflectance curve also cannot be fitted by the polynomial with a low degree (e.g., second degree) in a large range of wavelengths. However, in the O_2_-A absorption band, the shape of the reflectance curve within the range of 10 nm is close to a straight line; therefore, the accuracies of 3FLD and SFM basically do not change under the different parameters set used in this study.

The above analyses are based on data for which the chlorophyll content is 40 and the leaf area index is 4. However, for data with other leaf and canopy parameters, the results are essentially not different. Nonetheless, some details are different; for example, the degree of change in the accuracy of each algorithm under different parameters set is different due to the different shape of the reflectance near the oxygen absorption band, especially for FLD and 3FLD. The slope of the reflectance near the oxygen absorption band positively correlates with the degree of change in the accuracy of FLD, and the curvature of the reflectance near the oxygen absorption band correlates with the degree of change in the accuracy of 3FLD.

#### 3.1.2. Analysis of Each Fluorescence Extraction Algorithm Using Data with Different Chlorophyll Contents

**Figure 6:**
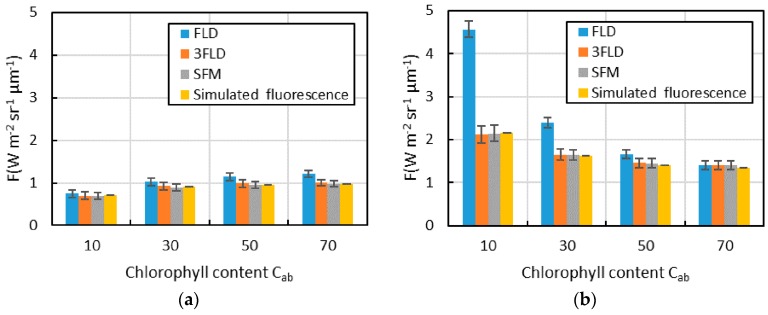
The fluorescence extraction results for each algorithm and the simulated value of fluorescence for data with different chlorophyll contents (the rectangle in this Figure is the mean fluorescence, and the error lines are the standard deviation values); (**a**) O_2_-A absorption band; (**b**) O_2_-B absorption band.

For data with different chlorophyll contents, the fluorescence extraction results of each algorithm and the simulated value of fluorescence in the O_2_-A and O_2_-B absorption bands are shown in Figure 6. FLD overestimates the fluorescence value of the vegetation with all types of chlorophyll content, and the slope of the reflectance curve in the O_2_-A absorption band gradually increases with the increase in chlorophyll content, resulting in a gradual increase in the ratio of overestimation. However, in the O_2_-B absorption band, the ratio of overestimation gradually decreases with an increase in chlorophyll content. The fluorescence extraction accuracy of 3FLD and SFM are relatively high and close to the simulated value of fluorescence. In addition, they are not materially influenced by the chlorophyll content. This result is fundamentally similar to that of Hu *et al.* [20]. However, some differences remain, such as changes in the accuracy of 3FLD in the O_2_-B absorption band.

In 3FLD, if the wavelengths on each side of the absorption band are far from the bottom of the absorption band, the accuracy is largely influenced by the curvature of the reflectance curve near the absorption band; therefore, the range of accuracy changing with chlorophyll content is very large. Thus, the change in fluorescence extraction accuracy with the change in chlorophyll content differs due to the different parameter settings. Moreover, with changes in the spectral resolution and spectral sampling interval, the parameter setting of each fluorescence extraction algorithm and the rule of accuracy changing with chlorophyll content may also change. In addition, the influence of noise on the standard deviation of the fluorescence value of each algorithm is very obvious in Figure 6, in which the standard deviations of FLD and 3FLD are slightly larger than that of SFM. If the data had not been smoothed, the difference would be larger.

#### 3.1.3. Analysis of Each Fluorescence Extraction Algorithm for Data with Different Leaf Area Indices

**Figure 7:**
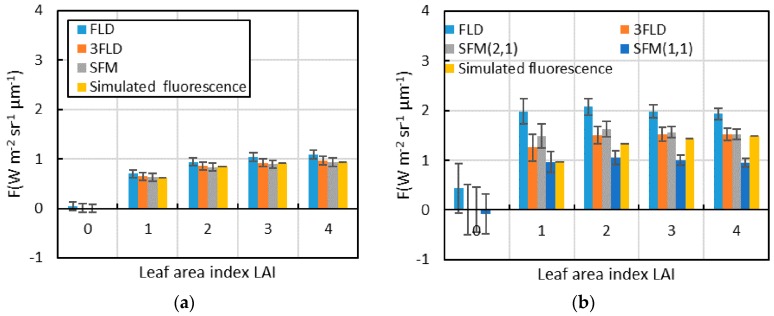
The fluorescence extraction results of each algorithm and the simulated value of fluorescence for data with different leaf area indices (the rectangle in this Figure is the mean fluorescence, and the error lines is the standard deviation); (**a**) O_2_-A absorption band; (**b**) O_2_-B absorption band.

For data with different leaf area indices, the parameter setting of each algorithm in the O_2_-A and O_2_-B absorption bands are basically same as shown in Table 3 and Table 4, and the fitting method that uses a linear function to fit the reflectance and fluorescence is newly increased in the parameter setting for SFM. The fluorescence extraction results of each algorithm and the simulated value of the fluorescence in the O_2_-A and O_2_-B absorption bands are shown in Figure 7. In the O_2_-B absorption band, the tendency is for the accuracy of each algorithm to increase with an increase in leaf area index. However, in the O_2_-A absorption band, the accuracy of each algorithm essentially does not change with a change in leaf area index, which is associated with variation in the curvature and slope of the trend line of the reflectance curve in the absorption band and also with the rate of fluorescence in the upward radiance. In addition, for the O_2_-B absorption band, when the leaf area index is relatively small, the reflectance curve is close to a straight line (as shown in Figure 3); therefore, the fluorescence extraction accuracy is relatively high when a linear function is used to fit reflectance and fluorescence (*i.e.*, SFM (1,1)). However, with an increase in leaf area index, the fluorescence extraction accuracy of SFM (1,1) decreases gradually. In contrast, the accuracy of using a quadratic function and a linear function to fit reflectance and fluorescence, respectively, (*i.e.*, SFM (2,1)) gradually increases and exceeds that of SFM (1,1) when the leaf area index reaches a certain number. For 3FLD, when the leaf area index is relatively small, the fluorescence value is overestimated under the parameter settings in this paper. Because 3FLD also assume the fluorescence to be a constant value and only change to assume the reflectance vary linearly comparing with FLD, similar to the parameters setting method of FLD analysed by Meroni *et al.* [16], appropriately increasing the wavelength on the left side (or right side) of the absorption band can improve the fluorescence extraction accuracy. Moreover, with a decrease in leaf area index, the standard deviation of the fluorescence value gradually increases because the radiance varies more rapidly than SNR. When the leaf area index and the actual fluorescence value are all relatively small, the influence of noise on the fluorescence extraction accuracy is very large.

#### 3.1.4. Analysis of Each Fluorescence Extraction Algorithm for Data with Different Solar Zenith Angles

**Figure 8:**
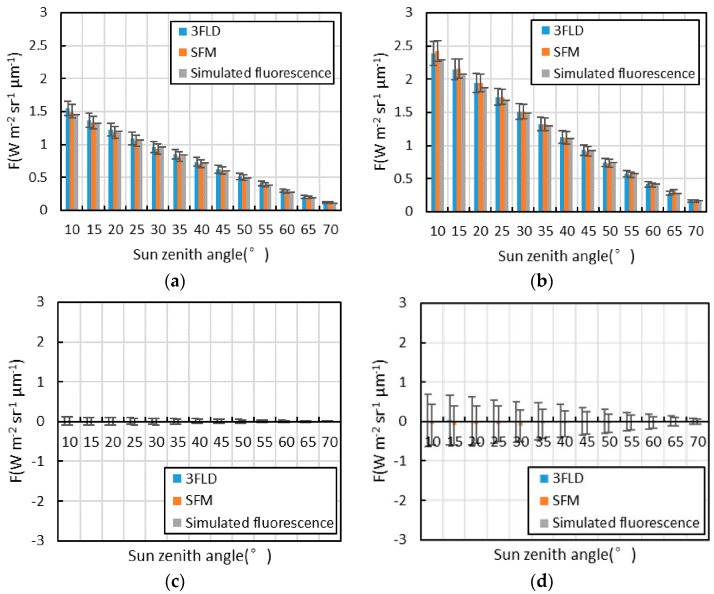
The fluorescence extraction results of each algorithm and the simulated value of fluorescence for data with different solar zenith angles (the rectangle in this Figure is the mean fluorescence, and the error lines is the standard deviation); (**a**) SIF of vegetation in the O_2_-A absorption band; (**b**) SIF of vegetation in the O_2_-B absorption band; (**c**) SIF of bare land in the O_2_-A absorption band; (**d**) SIF of bare land in the O_2_-B absorption band.

The fluorescence extraction results of each algorithm and the simulated value of fluorescence in the O_2_-A and O_2_-B absorption bands at different solar zenith angles are shown in Figure 8. With an increase in the solar zenith angle, the SIF of the vegetation decreases gradually. The SIF of bare land is much smaller than that of vegetation. Moreover, the radiances of vegetation and bare land decrease with an increase of solar zenith angle; therefore, the noise also decreases with an increase in solar zenith angle according to Equations (3) and (4), resulting in the standard deviation of SIF decreasing gradually. In the same way, the standard deviation of the SIF of bare land is larger than that of vegetation because the radiance of bare land in the O_2_-B absorption band is larger than that of vegetation. In addition, the O_2_-A absorption band is deeper than the O_2_-B absorption band. Therefore, the degree of sensitivity of SIF extraction accuracy in the O_2_-A absorption band is smaller than that in the O_2_-B absorption band, which results in the standard deviation of the SIF of bare land in the O_2_-A absorption band being much smaller than that in the O_2_-B absorption band. However, for vegetation, radiance in the O_2_-B absorption band is much smaller than that in the O_2_-A absorption band, and thus the noise in the O_2_-B absorption band is much smaller than that in the O_2_-A absorption band. The influence of the amplitude of the noise integrates with the influence of the depth of the absorption band, resulting in the difference between the vegetation SIF standard deviations in the O_2_-A and the O_2_-B absorption bands being much smaller than those of bare land.

### 3.2. Analysis of Measured Data

For the measured data, 3FLD and SFM are used to extract fluorescence in the O_2_-A and O_2_-B absorption bands, with relatively high accuracy in the analysis of the simulation data. In this process, the parameter settings of the simulation data are used to set the parameters for the measured data.

**Figure 9:**
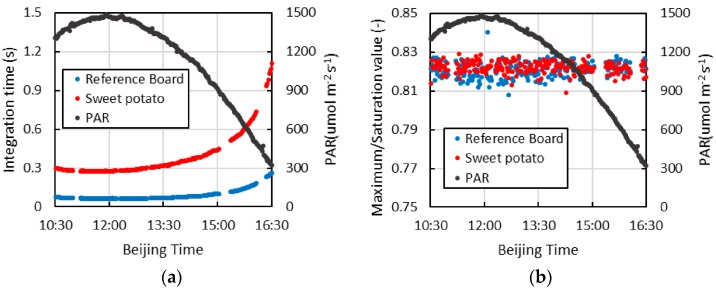
The integration time of using this continuous observation system to measure the spectrum of the reference plate and the canopy of sweet potatoes and the rate of maximum counts after optimizing the integration time to the saturated counts; (**a**) the integration time of measurement; (**b**) the rate of maximum counts after optimizing the integration time to the saturated counts.

Figure 9a is the integration time of using this continuous observation system to measure the spectrum of the reference plate and the canopy of sweet potatoes. Because the optimization method for integration time is adopted, the integration time of measuring the spectrum of the reference plate and canopy of sweet potatoes gradually increases with a decrease in PAR. Within the scope of the observation time, the integration time of measuring the spectrum of the reference plate is always less than 0.3 s, and that of measuring the spectrum of the canopy of sweet potatoes is always less than 1.2 s. With addition of the time of turning of the device-rotation motors (approximately 3~4 s) and the time of optimizing the integration time (less than the time of measuring the spectrum of the reference plate and canopy of sweet potatoes), from 10:30 A.M. to 4 P.M., the time of entire group of measurements can remain at approximately 5 s. Even after 4 P.M., when PAR decreases to 300 µmol·m^−2^·s^−1^, the measurement time for the entire group of can still be approximately 7 s. Therefore, this continuous observation system can decrease the uncertainty of fluorescence extraction caused by variation in illumination during the period of measurement.

Figure 9b is the rate of the maximum counts after optimizing the integration time to the saturated counts. Because the dark current is not deducted from the saturated counts in the process of optimizing the integration time, the practical counts are slightly larger than the expected value. During the entire measurement, the rate shown in Figure 9b is stable between 0.81 and 0.84. Therefore, the effect of this integration time optimization method is good. However, the sustained time of optimizing the integration time is far less than the iterative optimizing integration time method proposed by Cogliati *et al.* [6].

**Figure 10:**
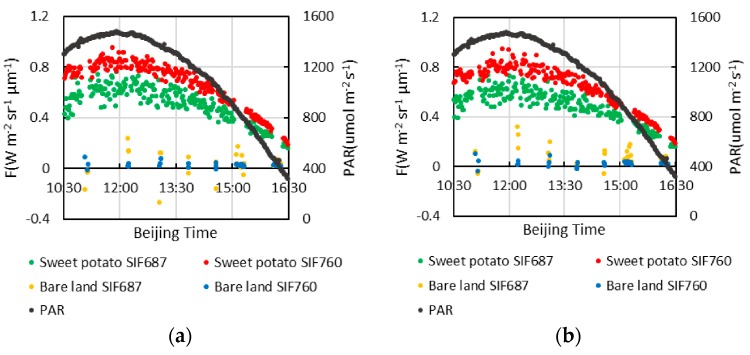
The diurnal variation in SIF at 687 nm and 760 nm of sweet potato and bare land extracted by 3FLD and SFM and the diurnal variation in PAR; (**a**) fluorescence extracted by 3FLD; (**b**) fluorescence extracted by SFM.

The SIF values at 687 nm and 760 nm (SIF687 and SIF760) of the sweet potato canopy and bare land extracted by 3FLD and by SFM and the diurnal variation curve of PAR are shown in Figure 10a,b. The fluorescence extraction results of these two algorithms are essentially the same, and the trend of the daily variation in SIF687 and SIF760 is basically the same as that of PAR. The value of SIF687 is smaller than that of SIF760, the size relationship of which is related to some factor such as the chlorophyll concentration, photosynthetic capacity or stress state [4]. The fluctuation in the diurnal variation curve of SIF687 and SIF760 results from environmental factors (e.g., rapid change in light, change in wind speed) or a change in physiology or noise. With the decrease in PAR, the fluctuation range of the diurnal variation curve of SIF also decreases gradually. Because the depth of the O_2_-B absorption band is much smaller than that of the O_2_-A absorption band, the fluorescence extracted in the O_2_-B absorption band is strongly affected by noise [16], which results in a relatively large fluctuation in the fluorescence extraction value. The SIF of bare land is much smaller than that of sweet potato in the same time period and is close to 0. Thus, the value extracted by this observation system is fluorescence rather than noise. However, the fluctuation in the SIF687 of bare land is much larger than that of SIF760, which is also related to the smaller depth of the O_2_-B absorption band. Moreover, the fluctuation in the SIF687 of bare land is much larger than that of vegetation, which is related to the fact that the noise of the bare land spectrum at this wavelength is larger than that of the vegetation spectrum according to Equations (3) and (4). Although the noise of the bare land spectrum in the O_2_-A absorption band is also very large, the depth of the O_2_-A absorption band is much larger than that of the O_2_-B absorption band. The influence of the noise to fluorescence value is therefore small, which results in the SIF760 fluctuation of bare land being relatively small. These results are essentially the same as those based on the simulated data, and the daily variation in SIF760 obtained by this observation system is essentially consistent with previous studies based on other continuous observation systems [6,7]. Therefore, the SIF extraction effect of this observation system is good. In addition, due to the high SNR and spectral resolution of the QE65pro spectrometer, this observation system can obtain SIF687 more accurately in comparison with other observation system. Previous studies have shown that the red to far-red F ratio is sensitive to stress and that it is beneficial to simultaneously collect red and the far-red F measurements [4,11]. In addition, this observation system can realize contrast observation of multiple targets, such as multiple vegetation in different stress states, which is conducive for analysing the change characteristics of fluorescence under stress.

**Figure 11:**
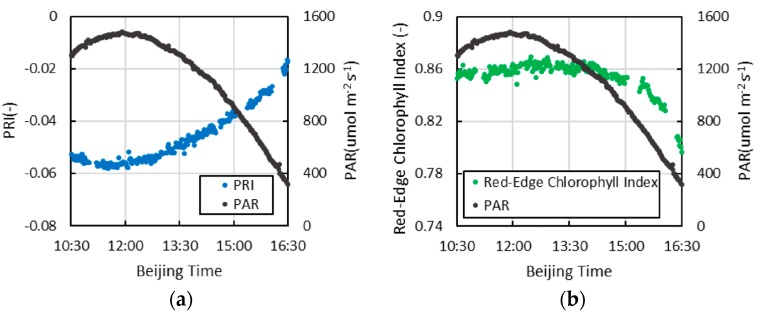
The diurnal variation in the Red-Edge Chlorophyll Index and PRI of sweet potato and the diurnal variation in PAR; (**a**) Red-Edge Chlorophyll Index; (**b**) PRI.

The diurnal variation in the Red-Edge Chlorophyll Index and PRI of sweet potato is shown in Figure 11. The former at local solar noon is not very large, only approximately 1%, but begins to accelerate markedly after 14:00. Moreover, the diurnal variation of PRI is relatively large at local solar noon compared to that of the Red-Edge Chlorophyll Index, with a variation trend that is opposite to that of SIF.

In the short term, the PRI value is linked to the xanthophyll cycle of the photo-protection mechanism at the leaf scale, which can reflect the state of photosynthesis [4]. Although the Red-Edge Chlorophyll Index is similar to the NDVI, it shows a linear relationship with the chlorophyll content and no saturation effect [21]. As the chlorophyll content often decreases under stress, the synchronous measurement of the SIF and vegetation index is very meaningful for researching the stress responses of vegetation.

## 4. Conclusions

To satisfy the demand for long-term comparative observation of different vegetation canopies, an automated comparative observation system for vegetation canopy SIF is designed in this study. This system is based on a QE65pro spectrometer and an MPM-2000 optical multiplexer, which has good expandability and can realize the comparative observation of multiple features. The spectral response range of this system is 475.9 to 862.2 nm, the spectral resolution is 0.9 nm, and the spectral sampling interval is 0.4 nm. This system not only can extract SIF in the O_2_-A and O_2_-B absorption bands but also can synchronously measure commonly used vegetation indices. To improve the SNR of the observed data, a type of single‑step optimization algorithm of integration time is proposed. This can rapidly optimize the integration time in each measurement, thus greatly reducing the influence on the results of fluorescence extraction from both random noise and irradiation variation between up-welling and down-welling measurements under cloudy conditions.

To optimize the accuracy of each fluorescence extraction algorithm, the vegetation canopy fluorescence model FluorMOD is used to simulate a set of simulated data that include different noise configurations, chlorophyll contents and leaf area indices according to the spectral sampling interval and SNR of the QE65pro spectrometer. This simulation method is similar to that used by Köhler *et al.* [22]. Based on these simulated data, the best parameter settings and accuracies of FLD, 3FLD and SFM under different conditions are summarized, and the degree of sensitivity of each algorithm to noise is also analysed. Through such analyses, 3FLD and SFM are confirmed to be suitable algorithms for extracting SIF from the spectral field measurements using the system proposed in this paper.

In field spectral measurement, 3FLD, SFM and their optimal parameters tested from simulated data are used to extract SIF values in O_2_-A and O_2_-B absorption bands from the sweet potato canopy and bare land, and the reflectivity spectral curve is used to calculate the Red-Edge Chlorophyll Index and PRI of the sweet potato canopy. The experiment shows that the results of field measurements are essentially the same as those for the simulated data, with the daily variation trend of the vegetation canopy SIF being essentially the same as that for PAR. The SIF of bare land is close to 0 and much smaller than that of vegetation. The above results verify the reliability of SIF in O_2_-A and O_2_-B absorption bands and the comparative ability of this observation system. Moreover, this system can synchronously obtain some commonly used vegetation indices such as the Red-Edge Chlorophyll Index and PRI, which is beneficial for comprehensively analysing the stress response characteristics of the vegetation canopy.

## Figures and Tables

**Table 1:** Parameters of the vegetation spectrum with different chlorophyll contents simulated by FluorMOD.

Input Parameter of FluorMOD	Parameter Value
The concentration of chlorophyll a + b (C_ab_, μg/cm^2^)	10, 30, 50, 70
leaf area index (LAI)	4

**Table 2:** Parameters of the vegetation spectrum with different leaf area indices simulated by FluorMOD.

Input Parameter of FluorMOD	Parameter Value
The concentration of chlorophyll a + b (C_ab_, μg/cm^2^)	40
leaf area index (LAI)	0, 1, 2, 3, 4

**Table 3:** Different parameters set of each fluorescence extraction algorithm for the O_2_-A absorption band.

	λ_in_	λ_left_	λ_right_	Degree of Polynomial (r, f)
FLD	760.519	757.282, 755.121, 753.319		
3FLD	760.519	756.201, 751.877, 750.072	765.189, 769.135, 771.284	
SFM	760.519	756.201, 754.040, 752.237	771.284, 773.074, 775.220	1, 1

**Table 4:** Different parameters set of each fluorescence extraction algorithm for the O_2_-B absorption band.

	λ_in_	λ_left_	λ_right_	Degree of Polynomial (r, f)
FLD	687.276	684.321, 682.102, 680.251		
3FLD	687.276	684.321, 682.442, 680.251	690.229, 692.441, 694.652	
SFM	687.276	685.06, 681.362, 679.141	690.229, 694.283, 696.124	2, 1

**Table 5:** The settings for each parameter in the O_2_-A absorption band.

	λ_in_	λ_left_	λ_right_	Polynomial Degree (r, f)
FLD	760.519	759.081		
3FLD	760.519	754.040	767.342	
SFM	760.519	758.721	768.776	1, 1

**Table 6:** The settings for each parameter in the O_2_-B absorption band.

	λ_in_	λ_left_	λ_right_	Polynomial Degree (r, f)
FLD	687.276	686.538		
3FLD	687.276	686.538	688.015	
SFM	687.276	683.581	691.704	2, 1
